# Surgical Versus Conservative Treatment of Post-Sternotomy Mediastinitis: Clinical Characteristics, Microbiology, and Outcomes from a 10-Year Cohort

**DOI:** 10.3390/diagnostics16050785

**Published:** 2026-03-06

**Authors:** Francesca Serapide, Lavinia Berardelli, Girolamo Perrotta, Simona Mongiardi, Antonio Di Virgilio, Giuseppe Musolino, Giuseppe Filiberto Serraino, Pasquale Mastroroberto, Alessandro Russo

**Affiliations:** 1 Infectious and Tropical Diseases Unit, “Renato Dulbecco” Teaching Hospital, Viale Europa, 88100 Catanzaro, Italy; lavinia.berardelli@gmail.com (L.B.); simonamongiardi@gmail.com (S.M.); a.russo@unicz.it (A.R.); 2 Department of Medical and Surgical Sciences, “Magna Graecia” University of Catanzaro, Viale Europa, 88100 Catanzaro, Italy; 3 Department of Experimental and Clinical Medicine, “Magna Graecia” University of Catanzaro, Viale Europa, 88100 Catanzaro, Italy; girolamo.perrotta@studenti.unicz.it (G.P.); serraino@unicz.it (G.F.S.); mastroroberto@unicz.it (P.M.); 4 Cardiac Surgery Unit, “Renato Dulbecco” Teaching Hospital, Viale Europa, 88100 Catanzaro, Italy; anto60divirgilio@gmail.com (A.D.V.); giuseppemusolino@hotmail.it (G.M.)

**Keywords:** mediastinitis, post-surgical complications, cardiac infections, cardiac surgery, chest infections

## Abstract

**Background**: Post-sternotomy mediastinitis (PSM) remains one of the most serious complications of cardiac surgery. This study aimed to evaluate the clinical features, management strategies, and outcomes of patients with PSM, comparing surgical and conservative treatment approaches. **Methods**: We retrospectively reviewed all cases of PSM (March 2014–July 2024) at our tertiary referral centre. **Results**: A total of 81 patients were included (39 surgically treated, 42 conservatively managed). The length of hospital stay was significantly longer in the surgical group (*p* = 0.003), and blood transfusions were more frequent (*p* = 0.005). Patients in the conservative group had higher DSW-STS risk scores (*p* = 0.014). Positive blood cultures were significantly more common among surgically treated patients (*p* < 0.001). The in-hospital mortality rate was 2.5% overall, with no difference between groups. **Conclusions**: These results likely reflect the greater clinical severity and complexity of patients selected for surgery, rather than an adverse effect of the procedure itself. Surgical treatment of PSM is associated with longer hospitalisation and greater need for blood transfusion, reflecting the higher clinical complexity of these cases. Nevertheless, outcomes in terms of survival were comparable to conservative management, supporting an individualised, multidisciplinary approach to optimise care for patients with post-sternotomy mediastinitis.

## 1. Introduction

Post-sternotomy mediastinitis (PSM) is a deep infection of the mediastinal tissues that occurs following median sternotomy and represents one of the most serious complications [1]. Although it occurs rarely in approximately 0.5–5% of patients undergoing cardiac surgery [1,2,3], it is associated with significant morbidity and mortality, often requiring intensive care management [4]. Furthermore in-hospital mortality ranges from 19 to 29% [5,6,7].

The pathogenesis of PSM is multifactorial. Patient-related factors, such as comorbidities, and characteristics of the procedure itself can compromise the wound healing process and increase the risk of infection [8]. Microbiologically, *S. aureus* and coagulase-negative staphylococci continue to be the predominant pathogens, although Gram-negative bacteria and polymicrobial infections are increasingly recognised [2]. Management of PSM requires an integrated surgical and medical strategy which should be assessed in relation to patient’s intrinsic and extrinsic characteristics [1]. The main surgical strategies encompass a range of revision methods like open dressings or closed irrigation, primary closure, or reconstruction with vascularised soft tissue flaps such as the omentum or pectoralis muscle [9,10]. Some of these are associated with high mortality and morbidity rates, prolonged hospital stays, and a significant risk of recurrence [11]. The introduction of vacuum-assisted closure therapy (VAC), a drainage system that applies negative pressure to the wound while stabilising the chest, has transformed the management of mediastinitis, significantly reducing mortality rates, hospital stays and complications [12,13,14,15]. However, despite progress in developing advanced dressing techniques (VAC) and tailored medical treatment approaches, there are still no definitive clinical practice guidelines, so management remains based on multidisciplinary consultations [4]. Given the significant impact of PSM on patient outcomes and healthcare resources, a better understanding of its clinical course and management outcomes remains essential. The aim of this study was to describe patient characteristics, microbiological profiles, and outcomes, and to compare surgical versus conservative treatment strategies. The study examines and compares the epidemiological and clinical characteristics, clinical outcomes and survival rates of patients with confirmed mediastinitis after cardiac surgery who were treated surgically or conservatively.

## 2. Materials and Methods

### 2.1. Study Design and Population

We conducted a retrospective single-centre study that included all adult hospitalised patients who developed PSM following cardiac surgery between March 2014 and July 2024 at our teaching referral hospital “Renato Dulbecco” in Catanzaro, Southern Italy. We enrolled all patients aged 18 years or older who met the following criteria: had undergone cardiac surgery with median sternotomy as the first intervention (valve replacement/implantation, myocardial revascularisation, thromboendoarterectomy, atrial myxoma exeresis), with a subsequent confirmed mediastinitis in accordance with clinical, radiological, and microbiological criteria of the Centers for Diseases Control and Prevention (CDC) definitions; had undergone revision surgery or conservative treatment. Patients younger than 18 years of age without evidence of deep mediastinal involvement were excluded. The necessity for written informed consent was superseded by the ethical committee on account of the observational and retrospective nature of the study and was conducted in accordance with Declaration of Helsinki.

### 2.2. Data Collection and Definitions

The study investigators collected patient data from medical records of the Cardiac Surgery Unit, the hospital’s computerised database, and/or medical file using a standard form. They used a predefined case report form (CRF) whose data was reported in a single database. Data collected in CRF included: demographics characteristics (age, sex comorbidities), risk factors (diabetes, obesity, smoking, chronic respiratory or renal disease) and preoperative variables. Surgical details included the type of cardiac procedure, timing (elective, urgent, or emergency), use of extracorporeal circulation (ECC), and bilateral internal mammary artery (BIMA) harvesting. Microbiological data were obtained from intraoperative wound swabs and blood cultures performed at diagnosis and during follow-up. The antibiotic regimens administered before and after microbiological results were also reviewed. A comparison was made between patients with confirmed mediastinitis following cardiac surgery treated with either a surgical approach or a conservative approach. Mediastinitis was defined according to CDC criteria and required at least one of the following: purulent discharge from the mediastinal space; isolation of pathogens from mediastinal tissue or fluid; evidence of mediastinal widening or abscess on imaging; clinical signs of infection (fever, leucocytosis) associated with sternal instability [16]. Length of hospital stay (LOS) was defined as the number of days from the index cardiac surgery to hospital discharge. Early mortality was defined as in-hospital death within 30 days of diagnosis.

### 2.3. Treatment Strategies

Surgical management consisted of aggressive debridement of infected or necrotic tissue, sternal rewiring or reconstruction when feasible, and vacuum-assisted closure therapy (VAC) as indicated. Antibiotic therapy was administered empirically upon diagnosis and subsequently tailored to microbiological results. Conservative treatment included systemic antibiotic therapy, local wound care, close clinical monitoring and vacuum-assisted closure therapy (VAC) as indicated. In all cases, management was decided by a multidisciplinary team involving cardiac surgeons, infectious disease specialists, and microbiologists.

The wound healing process was facilitated by vacuum therapy, which was considered an additional treatment in both groups.

### 2.4. Microbiological Methods

Gram staining and a rapid identification protocol were employed on positive culture samples. The bacterial pellet obtained directly from these cultures was processed for identification via MALDI-TOF MS (Bruker Daltonics, Billerica, MA, USA) and subsequently used for molecular testing. Antimicrobial susceptibility was assessed using either the SensiTitre™ system (Thermo Fisher Scientific, Waltham, MA, USA) or the Vitek 2 automated platform (bioMérieux, Marcy l’Etoile, France). Minimum inhibitory concentrations (MICs) were interpreted in accordance with the European Committee on Antimicrobial Susceptibility Testing (EUCAST) breakpoints. Starting from 2018, a multiplex PCR technique integrated with array-based detection was applied through an automated closed system (FilmArray, BioFire Diagnostics, bioMérieux, Salt Lake City, UT, USA), enabling simultaneous extraction, amplification, and identification of nucleic acids from a single specimen to detect pathogens and associated resistance genes.

### 2.5. Statistical Analysis

Continuous variables were expressed as mean ± standard deviation (SD) and categorical variables as absolute numbers and percentages. Differences between the surgical and conservative groups were assessed using the Welch *t*-test for continuous variables and the Chi-square or Fisher’s exact test for categorical variables, as appropriate. A two-sided *p* < 0.05 was considered statistically significant. Statistical analysis was performed using aggregated summary data, given the retrospective design.

Logistic regression was used to assess associations, providing odds ratios (OR), 95% confidence intervals (CI), and *p*-values.

The level of statistical significance was set at 0.05 for all tests. All statistical analyses were conducted using Stata 18.

## 3. Results

### 3.1. Patients’ Characteristics

During the study period a total of 81 patients who met the inclusion criteria were enrolled. The patients were divided into two groups: patients undergoing surgical wound revision (39/81; 48.1%) and patients undergoing conservative therapy (42/81; 51.9%). Baseline demographic and clinical characteristics are reported in Table 1.

### 3.2. Clinical and Demographic Features

The mean age was slightly higher in the surgical group (66.7 ± 8.4 years) than in the conservative group (62.8 ± 10.6 years), although this difference did not reach statistical significance (*p* = 0.107). There were no significant differences in the prevalence of major comorbidities such as diabetes, hypertension, or obesity. However, a history of previous cardiac surgery was more frequent among patients undergoing surgical management (33.3% vs. 14.3%, *p* = 0.043). The mean length of hospitalisation was significantly longer in the surgical group (60.1 ± 40.4 days vs. 37.7 ± 20.9 days, *p* = 0.003).

### 3.3. Surgical and Perioperative Features

Operative data and perioperative variables are summarised in Table 2. The most common surgical indication was coronary artery bypass grafting (CABG)**,** performed in 74.1% of the surgical group and 83.3% of the conservative group (*p* = 0.267). The use of cardiopulmonary bypass (ECC) and BIMA harvesting did not differ significantly between groups. However, blood transfusions were required more frequently in the surgical group (87.2% vs. 59.5%, *p* = 0.005). Patients in the conservative group presented significantly higher DSW-STS scores (3.4 ± 4.0 vs. 1.7 ± 1.6, *p* = 0.014), reflecting greater operative risk.

### 3.4. Microbiological Findings

Microbiological results are shown in Table 3.

Coagulase-negative staphylococci and *S. aureus* were the most frequently isolated pathogens from wound swabs in both groups. Polymicrobial infections occurred in 44.8% of surgical patients and 27.3% of conservatively managed ones (*p* = 0.205). Notably, **positive blood cultures** were significantly more frequent in the surgical group (33.3% vs. 4.8%, *p* < 0.001), while *Candida* spp. were isolated only among surgical patients.

### 3.5. Antimicrobial Therapy

Antimicrobial regimens are detailed in Table 4.

Almost all patients received targeted antimicrobial therapy. Combination treatment was used in approximately half of cases (51.3% vs. 40.5%, *p* = 0.341). β-lactam antibiotics were significantly more frequently administered in the surgical group (28.2% vs. 7.1%, *p* = 0.012). VAC was widely employed in both groups (76.9% vs. 57.1%, *p* = 0.072).

### 3.6. Clinical Outcomes

Table 5 summarises the primary and secondary outcomes stratified by treatment group.

Overall, short-term survival was excellent and did not differ significantly between groups. The 14-day survival rate was 97.5% (38/39 surgical vs. 41/42 conservative, *p* = 0.482). Negative wound swabs during follow-up were observed in 55.2% of surgical and 54.5% of conservative patients (*p* = 0.958). In-hospital mortality occurred in only one patient per group (*p* = 0.958).

### 3.7. Multivariate Analysis

Table 6 reports the multivariate analysis of variables associated with the surgical approach versus conservative treatment; the only factor independently associated with surgical approach was transfusion (OR 3.75, CI 95% 1.05–13.42, *p* = 0.042) (Table 6). In fact, patients who underwent transfusion were approximately four times more likely to undergo surgical revision.

However, no factor was significantly associated with mortality in this patient population.

## 4. Discussion

PSM remains a severe complication after cardiac surgery, despite advances in prevention, early diagnosis, and multidisciplinary management. Reported incidence varies from 0.5% to 5% [1,2,3], with mortality between 19% and 29% [5,6,7]. This study reviews PSM cases over ten years in a tertiary referral centre, comparing surgical and conservative approaches. In our analysis, based on CDC criteria [16], mediastinitis occurred in 2.4% of 3354 sternotomies in the study period. The rate at our centre is lower than most reports, and early mortality was only 2.5%, likely due to prompt diagnosis, multidisciplinary care, and surgical expertise. The mean patient age was 64.6 years, 67.9% were male, and common comorbidities included hypertension, diabetes, obesity, and smoking. These characteristics are consistent with those reported in the extant literature [11,17,18]. Our analysis showed that patients undergoing surgical treatment experienced a significantly longer hospital stay and more frequent need for blood transfusion than those managed conservatively. This likely reflects the greater clinical severity and complexity of patients selected for surgery, rather than an adverse effect of the procedure itself. Importantly, early survival outcomes did not differ between groups, supporting the concept that both strategies can be effective when appropriately tailored to the individual case. Furthermore, whilst the duration of stay was found to be shorter in many studies, it was confirmed that a conservative approach is associated with a reduced length of hospitalisation [19]. This improvement is likely attributable to enhanced patient management, the incorporation of innovative devices, and a reduced probability of complications that could prolong patient stays. Higher DSW-STS scores in the conservative group indicate that these patients were considered at greater operative risk, leading clinicians to prefer non-surgical management. Conversely, the higher rate of positive blood cultures in the surgical group probably mirrors more extensive infections, with a higher bacterial burden and systemic dissemination prompting surgical debridement.

### 4.1. Microbiology and Antibiotic Therapy

Consistent with previous studies [5,20,21], as well as the most recent observational study involving a cohort of 900 patients stratified by three treatment groups [3], *S. aureus* and coagulase-negative staphylococci (CNS) were the most common pathogens identified. Gram-negative bacteria and polymicrobial infections were also frequent, confirming the evolving microbiological landscape of PSM highlighted in the recent literature [22,21]. Notably, *Candida* spp. was isolated exclusively among surgically treated patients, underlining the importance of broad microbiological screening and antifungal coverage in selected cases. The pattern of antimicrobial use reflected both empirical and targeted strategies. β-lactams were significantly more common among surgical patients, possibly due to their role in perioperative prophylaxis and in managing Gram-positive cocci.

From a therapeutic standpoint, except for perioperative regimens (beta-lactams and occasionally carbapenems) employed in accordance with hospital surgical protocols, empirical treatments were informed by local epidemiology, with the identification of methicillin-resistant staphylococci. In fact, in line with the extant literature, staphylococci isolated from wounds and blood cultures were found to be resistant to methicillin, necessitating the predominant use of drugs such as vancomycin, linezolid, or daptomycin, which are often difficult to manage in this category of patients. This factor should prompt reflection on the challenging management of PSM, not only in surgical terms, but also in terms of antimicrobial resistance, which poses an additional challenge for clinicians and necessitates the integration of a multidisciplinary team.

Despite the absence of statistically significant differences in antifungal or combination therapy, antimicrobial stewardship remains crucial, as prolonged or empirical treatments may increase resistance and toxicity.

### 4.2. Clinical Implications

The comparable survival between surgical and conservative groups supports the current trend towards an individualised approach, balancing surgical risk with infection severity. Surgery remains the mainstay of treatment for deep or extensive mediastinal infections, particularly when associated with sepsis, sternal instability, or persistent bacteraemia. Conservative management, combining targeted antibiotic therapy and meticulous wound care, may represent a valid option in selected high-risk or frail patients, especially when the infection is localised and the patient remains haemodynamically stable. VAC was widely employed across both groups in our cohort. Negative pressure wound therapy has revolutionised the management of PSM, promoting granulation, improving local perfusion, and reducing bacterial load. The absence of a statistically significant difference in its use between groups likely reflects its widespread adoption as the standard of care.

This study has several limitations. First, its retrospective nature and the use of aggregated data limit the ability to perform multivariate analyses to control for potential confounders while also hampering the performance of certain statistical analyses, such as corrections for baseline inferences between the two groups, thereby limiting their interpretation in some cases. Additionally, as the study design is limited to a description of all patients treated at our cardiac surgery centre who developed PSM, there is a lack of statistical power, leading again to limitations on the interpretation of the results. Second, microbiological data were based on available culture results, and molecular methods were not systematically employed. Third, long-term follow-up beyond hospital discharge was not available, preventing assessment of recurrence or late mortality. Finally, the choice between surgical and conservative treatment was not randomised but guided by clinical judgement of the treating physicians, introducing an important selection bias. Furthermore, the nature of the study limited the possibility of acquiring long-term outcomes (recurrence, late mortality, functional status).

Despite these limitations, this study provides one of the most extensive single-centre analyses of PSM management for over more than a decade, integrating clinical, microbiological, and therapeutic data. The inclusion of both surgical and conservatively treated patients allows a real-world comparison of strategies, contributing valuable evidence to guide multidisciplinary decision-making.

In conclusion, PSM remains a severe but manageable complication of cardiac surgery when addressed within a multidisciplinary framework. Surgical treatment is associated with longer hospitalisation and greater transfusion requirements, reflecting higher clinical complexity, whereas conservative management is more often chosen for high-risk or frail patients. Despite these differences, survival outcomes were comparable between the two groups, underscoring the importance of individualised treatment selection rather than a one-size-fits-all approach.

Early diagnosis, close collaboration between cardiac surgeons, infectious disease specialists, and microbiologists, and the judicious use of antimicrobial therapy are key to optimising patient outcomes.

Future prospective, multicentre studies are warranted to better define selection criteria for surgical versus conservative management and to evaluate the long-term impact of different therapeutic strategies on morbidity, mortality, and quality of life.

## Figures and Tables

**Table 1:** Clinical and demographic features of patients with post-surgical mediastinitis stratified by treatment group.

Variables	Total *N* = 81	Surgical Approach Group *N* = 39	Conservative Approach Group *N* = 42	*p*-Value
**Anamnestic factors**				
Age (years) ± SD	64.6 ± 9.8	66.7 ± 8.4	62.8 ± 10.6	0.069
Male sex	55 (67.9)	25 (64.1)	30 (71.4)	0.48
Pre-surgery hospitalisation (days) ± SD	12.3 ± 10.1	14.1 ± 11.8	10.7 ± 7.9	0.135
Length of Hospitalisation (days)	48.5 ± 33.7	60.1 ± 40.4	37.7 ± 20.9	**0.003**
**Comorbidities**				
Diabetes	40 (49.4)	17 (43.6)	23 (54.8)	0.315
Obesity	17 (21)	7 (19)	10 (26.8)	0.517
Hypertension	65 (80.2)	32 (82.0)	33 (78.6)	0.694
Smoke	32 (39.5)	13 (33.3)	19 (45.2)	0.273
COPD	11 (13.6)	7 (17.9)	4 (9.5)	0.269
CRF	10 (12.3)	5 (12.8)	5 (11.9)	1.0
Malignancies	4 (4.9)	3 (7.7)	1 (2.4)	0.347
Stroke	8 (9.9)	4 (10.2)	4 (9.5)	1.0
Hematologic disorders	1 (1.2)	1 (2.6)	0 (0)	0.481
Polypharmacy	63 (77.7)	30 (76.9)	33 (78.6)	0.858
Recurrent infections	2 (2.5)	1 (2.6)	1 (2.4)	1.0
Prosthetic devices	4 (4.9)	3 (7.7)	1 (2.4)	0.347
Heart failure	10 (12.3)	5 (12.8)	5 (11.9)	1.0
Previous cardiac surgeries	19 (23.4)	13 (33.3)	6 (14.3)	**0.043**

**Legend:** COPD: Chronic Obstructive Pulmonary Disease; CRF: Chronic Respiratory Failure.

**Table 2:** Initial surgical approaches for patients stratified by treatment group.

Variables	Total *N* = 81	Surgical Approach Group *N* = 39	Conservative Approach Group *N* = 42	*p*-Value
**Surgical technique**				
Myocardial revascularisation	70 (86.4%)	33 (84.6%)	37 (88.1%)	0.648
Isolated CABG	64 (91.4%)	29 (87.9%)	35 (94.6%)	0.322
CABG + Valve	6 (8.6%)	4 (12.1%)	2 (5.4%)	0.421
Valvuloplasty and/or valve replacement	10 (12.3%)	4 (10.2%)	6 (14.3%)	0.739
Other surgeries	3 (3.7%)	2 (5.1%)	1 (2.4%)	0.606
**Timing of surgery**				
Non-urgent	71 (87.6%)	34 (79.5%)	37 (88.1%)	1.0
Emergency	6 (7.4%)	2 (5.1%)	4 (9.5%)	0.677
Urgent	4 (4.9%)	3 (7.7%)	1 (2.4%)	0.347
**Antimicrobial prophylaxis**	58 (71.6%)	31 (79.5%)	27 (64.3%)	0.13
Cefazolin	33 (56.9%)	15 (48.4%)	18 (66.7%)	0.161
Vancomycin	1 (1.7%)	1 (3.2%)	0 (0)	1.0
Cefazolin plus vancomycin	18 (31.0%)	12 (38.7%)	6 (22.2%)	0.176
Others	6 (10.4%)	3 (9.7%)	3 (11.1%)	1.0
BIMA (bilateral internal mammary artery)	15 (18.5%)	6 (15.4%)	9 (21.4%)	0.484
Intensive care unit hospitalisation in days (mean, ± SD)	3.2 ± 7.6	4.4 ± 10.6	2.0 ± 1.9	0.171
Orotracheal intubation duration in hours (mean ± SD)	2.7 ± 3.4	3.2 ± 2.5	2.3 ± 4	0.225
DSW-STS score (mean ± SD)	2.5 ± 3.2	1.7 ± 1.6	3.4 ± 4	**0.014**
ECC	62 (76.5%)	32 (82.0)	30 (71.4)	0.26
ECC duration in minutes (mean ± SD)	75.6 ± 51.3	79 ± 47.4	72.5 ± 54.5	0.568
Blood transfusion	59 (72.8%)	34 (87.2%)	25 (59.5%)	**0.005**
Blood bags (mean ± SD)	3.5 ± 3.9	4.4 ± 4.3	2.7 ± 3.4	0.053

**Legend:** CABG (coronary artery bypass grafting); BIMA (bilateral internal mammary artery); ECC (extracorporeal circulation).

**Table 3:** Microbiological isolates from wound swab and blood cultures stratified by treatment group.

Variables	Total *N* = 81 (%)	Surgical Approach Group *N* = 39 (%)	Conservative Approach Group *N* = 42 (%)	*p*-Value
***Surgical wound swabs***	***51 (63.0)***	***29 (74.3)***	***22 (52.4)***	
*Co-N Staphylococci*	30 (58.8)	16 (55.2)	14 (63.6)	0.543
*Staphylococcus aureus*	13 (25.5)	9 (31.0)	4 (18.2)	0.297
*Other Gram-positive bacteria*	11 (21.6)	5 (12.8)	6 (14.3)	0.498
*Enterobacterales*	9 (17.6)	7 (24.1)	2 (9.1)	0.268
*Other Gram-negative bacteria*	9 (17.6)	4 (13.8)	5 (22.7)	0.474
*Candida* spp.	3 (5.9)	3 (10.3)	0 (0)	0.249
*Polymicrobial isolation*	19 (37.2)	13 (44.8)	6 (27.3)	0.199
**Blood cultures**	***15 (18.5)***	***13 (33.3)***	***2 (4.8)***	**<** ***0.001***
*Co-N Staphylococci*	10 (66.7)	8 (61.5)	2 (100)	0.524
*Staphylococcus aureus*	1 (6.7)	1 (7.7)	0 (0)	1.0
*Enterobacterales*	1 (6.7)	1 (7.7)	0 (0)	1.0
*Other Gram-positive bacteria*	2 (13.3)	2 (15.4)	0 (0)	1.0
*Other Gram-negative bacteria*	2 (13.3)	2 (15.4)	0 (0)	1.0
*Polymicrobial isolation*	1 (6.7)	1 (7.7)	0 (0)	1.0

**Legend:** Co-N: coagulase-negative bacteria.

**Table 4:** Antibiotic therapy for patients with diagnosis of mediastinitis and VAC stratified by treatment group.

Variables	Total *N* = 81 (%)	Surgical Approach Group *N* = 39	Conservative Approach Group *N* = 42	*p*-Value
*Antimicrobial* *therapy*	*60 (74.0%)*	*32 (82.0%)*	*28 (66.6%)*	0.114
Linezolid	15 (25.0%)	9 (28.1%)	6 (21.4%)	0.309
Quinolones	19 (31.6%)	12 (37.5%)	7 (25.0%)	0.134
Sulfamethoxazole/trimethoprim	6 (10.0%)	1 (3.1%)	5 (17.8%)	0.203
Daptomycin	14 (23.3%)	9 (28.1%)	5 (17.8%)	0.184
Rifampicin	6 (10.0%)	3 (9.4%)	3 (10.7%)	1.0
Tigecycline	8 (13.3%)	4 (12.5%)	4 (14.3%)	1.0
Carbapenems	12 (20.0%)	7 (21.9%)	5 (17.8%)	0.444
β-lactams	14 (23.3%)	11 (34.4%)	3 (10.7%)	**0.012**
BL/BLI	7 (11.7%)	6 (18.7%)	1 (3.6%)	0.052
Glycopeptides	9 (15%)	4 (12.5%)	5 (17.8%)	1.0
Antifungal therapy	4 (6.7%)	2 (6.2%)	2 (7.1%)	1.0
Other	9 (15.0%)	4 (12.5%)	5 (17.8%)	1.0
Combination therapy	37 (61.7%)	20 (62.5%)	17 (60.7%)	0.329
*VAC-therapy*	*54 (66.7%)*	*30 (76.9%)*	*24 (57.1%)*	*0.059*

**Legend:** VAC—vacuum-assisted closure therapy, BLI: beta-lactam inhibitors.

**Table 5:** Primary and secondary outcomes stratified by treatment group.

Variable	Total (%) (*N* = 81)	Surgical Approach Group (%) (*N* = 39)	Conservative Approach Group (%) (*N* = 42)	*p*-Value
Survival at 14 days	79 (97.5)	38 (97.4)	41 (97.6)	1.0
Negative wound swabs	28 (54.9)	16 (55.2)	12 (54.5)	0.964
Negative blood cultures	4 (26.6)	4 (30.8)	0 (0)	1.0
In-hospital mortality	2 (2.5)	1 (2.6)	1 (2.4)	1.0

**Table 6:** Multivariate analysis of factors associated with surgical approach.

Variable	OR	IC 95%	*p*-Value
Age (years)	1.04	0.99–1.10	0.13
Length of hospital stay (days)	1.04	0.98–1.09	0.19
Extracorporeal circulation	2.90	0.37–22.64	0.31
Length of EC (minutes)	0.99	0.97–1.01	0.19
**Transfusions**	**3.75**	**1.05–13.42**	**0.042**
Score DSW	1.00	0.97–1.03	0.89

**Legend:** EC: extracorporeal circulation.

## Data Availability

The data presented in this study are available on request from the corresponding author due to privacy reason.

## Abbreviations

The following abbreviations are used in this manuscript:

COPD	Chronic Obstructive Pulmonary Disease
CRF	Chronic Respiratory Failure; CABG (coronary artery bypass grafting)
BIMA	(Bilateral internal mammary artery)
ECC	(Extracorporeal circulation)
Co-N	Coagulase-Negative bacteria
VAC	Vacuum-Assisted Closure Therapy
BLI	Beta-lactam inhibitors
EC	Extracorporeal circulation
